# Assessing methods for estimating microbial lag phase duration: a comparative analysis using *Saccharomyces cerevisiae* empirical and simulated data

**DOI:** 10.1093/femsyr/foaf033

**Published:** 2025-07-11

**Authors:** Monika Opalek, Dominika Wloch-Salamon, Bogna J Smug

**Affiliations:** Institute of Environmental Sciences, Faculty of Biology, Jagiellonian University, 31-007 Krakow, Poland; Institute of Environmental Sciences, Faculty of Biology, Jagiellonian University, 31-007 Krakow, Poland; Malopolska Centre of Biotechnology, Jagiellonian University, 31-007 Krakow, Poland

**Keywords:** lag phase length, microbial growth curve, microbial growth kinetics, fitness, lag time, bacterial growth models

## Abstract

The lag phase is a temporary, nonreplicative period observed when a microbial population is introduced to a new, nutrient-rich environment. Although the theoretical concept of growth phases is clear, the practical application of methods for estimating lag lengths is often challenging. In fact, there are two distinct assumptions: (i) that cells do not divide at all during the lag phase or (ii) that they divide but at a suboptimal rate. Therefore, the choice of method should consider not only technical limitations but also consistency with the biological context. Here, we investigate the performance of the most common lag estimation methods, using empirical and simulated datasets. We apply different biological scenarios and simulate curves with varying parameters (i.e. growth rate, noise level, and frequency of measurements) to test their impact on the estimated lag phase duration. Our validation shows that infrequent measurements, low growth rate, longer lag phases, or higher level of noise in the measurements result in higher bias and higher variance of lag estimation. Additionally, in case of noisy data, the methods relying on model fitting perform best.

## Teaser

The effect of method assumptions (i.e. no cell divisions or divisions at a suboptimal rate) on the estimated lag phase lengths was investigated using empirical and simulated growth curves.

## Principles and concepts

While biological phenomena often follow logical and predictable patterns, there is always a certain degree of variability between individual organisms and populations. To analyse and interpret biological phenomena, it is necessary to apply some definitions and frameworks that allow objective comparisons. Such frameworks must simultaneously capture the pattern and its reproducibility, while at the same time allowing variability to be quantified (McEntire et al. 2021). While the theoretical concept of growth phases seems straightforward, defining growth phases accurately remains challenging due to the vast number of exceptions observed in biological systems. The complexity and variability within living organisms make it difficult to draw clear and definitive boundaries. As our knowledge grows, so does our recognition of the nuances and complications involved. Still, we need definitions to objectively compare and draw conclusions from biological data. For example, a robust analysis of the kinetics of multiple growth curves can help draw conclusions about the adaptive strategies of microbial populations under investigation.

In various scientific and industrial fields working with microorganisms, it is common practice to use growth curve kinetics analysis to describe microbial populations. Looking at the growth curve, we can easily identify distinct, reproducible growth phases, where each can be logically interpreted in the context of its significance and the underlying biological mechanisms. The typical progression of a microbial growth curve includes four distinct stages. First is the lag phase, where cells adjust to a new environment before divisions (at maximal rate). This is followed by the exponential (or log) phase, during which cells multiply at their maximum rate, causing the population size to double consistently. Next is the stationary phase (SP), which occurs when growth halts due to the exhaustion of essential nutrients. Finally, if observed for a longer duration, the decline or death phase emerges, characterized by a reduction in population size due to cellular death. It is important to remember that such definitions are always simplifications with inherent assumptions and limitations, serving as frameworks within which biological phenomena can be interpreted (Madigan et al. 2018). The most common laboratory method employed to acquire microbial growth curves is optical density measurements taken at intervals by a spectrophotometer.

Efficient analysis of microbial growth curves is crucial for various industrial settings. The food industry uses microbial growth curves to ensure food safety and quality, including e.g. predicting shelf life of perishable products (Dalgaard 1995, Chaturvedi et al. 2023), evaluating the effectiveness of food preservatives (Abi Assaf et al. 2024), or studying the growth of probiotic bacteria in fermented foods (Megur et al. 2023). In pharmaceutical research and production, microbial growth curves are used for e.g. optimizing antibiotic production and assessing the efficacy of antimicrobial compounds (Abi Assaf et al. 2024) or monitoring fermentation processes for drug production (Danquah and Forde 2008). In biotechnology, growth curve analysis is employed for optimizing conditions for biomass production and developing new fermentation processes for industrial products (Fu et al. 2012), including also alcoholic beverage industry, where growth curve analysis is used for monitoring yeast growth during fermentation and optimizing fermentation conditions for flavour development (Verbelen et al. 2009).

Advances in technologies allow for high-throughput experiments and industrial production lines, where multiple growth curves are analysed simultaneously. Thus, a well-defined and clear framework is essential for objective analysis and conclusions. To systematize and define the boundaries of successive growth phases, mathematical models are used that aim to accurately represent the shape of the growth curve. These models facilitate the standardization of analyses, enabling consistent and reproducible interpretations. However, building a model requires precise definitions and answers to the following questions: What is the lag phase? and How to detect the end of the lag phase? (Smug et al. 2024)

### What is the lag phase?

In broad terms, the lag phase is defined as the period during which microbial cells prepare for growth. Indeed, adjustment to a new environment after growth-arrest requires broad cellular reorganizations, which are universal for prokaryotes and eukaryotes (Bertrand 2019). The cells need to adjust their transcriptome and proteome [e.g. in *Saccharomyces cerevisiae* (Brejning and Jespersen 2002, Brejning et al. 2003, Brejning et al. 2005)] and rearrange cellular components that are necessary for nutrient uptake and biomass accumulation. These processes are activated within minutes after environment change [e.g. *Saccharomyces cerevisiae* (Martinez et al. 2004, Cucinotta et al. 2021) and *Salmonella enterica* (Rolfe et al. 2012)]. It is critical for the fitness of an organism to efficiently prepare for cell division, yet the duration of the lag phase is rarely used as a fitness marker.

Classically, fitness is defined by the number of progeny, which in microbiology is translated into the growth rate (i.e. the rate at which the population size doubles). While the exponential (maximum) growth rate is the most commonly used fitness measure (e.g. Jasnos et al. 2008), the quantification of the lag phase duration is equally important to assess the stress or fitness of microbial populations (Reding-Roman et al. 2017, Hamill et al. 2020). In fact, measuring the duration of the lag phase can sometimes provide a better estimation of microbial fitness than measuring the maximal growth rate. It is because the growth rate reflects the speed of replication and does not account for the efficiency of predivision cellular adaptations, such as the evolution to optimize lag duration in a fluctuating and stochastically changing environment (Himeoka and Mitarai 2021). When microbial population is exposed to stressful conditions such as extreme pH, temperature, or osmolarity, the duration of the lag phase can be a more sensitive indicator of fitness. Microbes that have previously experienced these kinds of stress tend to have shorter lag phases when re-exposed to the same conditions (while growth rate remains unaffected), indicating better fitness and adaptability (Vriesekoop and Pamment 2005, Cerulus et al. 2018, Vermeersch et al. 2019, Bheda 2020). Interestingly, this effect has also been observed in daughter cells that had never experienced the initially introduced conditions (Cerulus et al. 2018). Measuring lag phase duration can also be valuable to asses fitness based on populations ability to quickly transition from a nonreplicative to a replicative state, when introduced to new nutrient-rich environments (Opalek et al. 2022). The lag phase duration can be therefore a more direct measure of adaptive capability compared to the maximal growth rate, which only reflects the speed of replication once adaptation has occurred. Shorter lags enable earlier divisions, which allows to produce a higher number of progeny within a fixed period of time. This would confer a fitness advantage in a competition for limited resources. Thus, the short lag phase is generally believed to be beneficial, and therefore, populations in favourable conditions may be expected to evolve towards decreased lag duration (Adkar et al. 2017). However, the opposite strategy was observed in the presence of antibiotics. In particular, when bacterial populations were exposed to antibiotics before a transfer to new growth media, they evolved towards increased lag duration which matched the duration of antibiotic exposure. This strategy minimized the toxic effects of antimicrobial exposure (Fridman et al. 2014). More broadly, the concept of evolution to optimize the ‘wake-up’ strategy under antibiotic exposure has been investigated by Himeoka and Mitarai (2021).

### How to detect the end of the lag phase? Assumptions and models

From the perspective of an individual cell observed under a microscope, the end of the lag phase is often associated with the first morphological signs of bud formation (e.g. Lee et al. 2016, Cerulus et al. 2018, Moreno-Gámez et al. 2020).

Within the studies at the population level, the lag phase can be defined as the time before any detectable increase in the cell abundance (biomass) (Opalek et al. 2022), or as the time delay before a population reaches exponential growth (Rolfe et al. 2012). Biologically, the population-level lag phase is a combination of two subsequent physiological processes: a no-growth phase that turns into suboptimal growth, when cells initiate divisions but have not yet reached the full growth rate. It is often not possible to separate these processes efficiently using standard laboratory methods such as spectrophotometry.

This distinction is also incorporated in the assumptions of the methods used to estimate the duration of the lag phase from the growth curve. The simplest method that assumes no growth in the lag phase is the ‘biomass increase’ method, which identifies the end of the lag phase as the point at which the biomass has increased from the initial value by a predefined threshold (usually a minimum detectable increase) (Opalek et al. 2022). The most frequently used method of calculating the lag duration (Jomdecha and Prateepasen 2011, Cerulus et al. 2018, Valík et al. 2021) is the ‘tangent method’ (Bertrand 2019). It determines the end of the lag phase as the intersection between the tangent line to the point of maximum growth rate and the *y* = log(*N*0) line, where *N*0 is the inoculation density. It provides an easily interpretable result under the assumption that there is no population growth during the lag phase and that cells then begin to divide synchronously at a constant growth rate. It can also be applied for growth curves where cells divide at a suboptimal rate during the lag phase, but the interpretation is not clear. Specifically, the method determine a lag phase as a time point between any growth beginning and before the maximum growth rate of the population is reached. The ‘max growth acceleration’ method, where the end of the lag phase is defined as the point of the growth curve where the second derivative of the population size in time is maximal (Buchanan and Cygnarowicz 1990, Liu et al. 2021), can be applied under both assumptions (no growth or suboptimal growth during the lag phase). This will be further discussed within the ‘Testing methods for lag phase duration determination on the model-simulated dataset’ section. As for the method of fitting the entire growth curve to the model, the definition of lag is related to the model chosen. While the logistic model (fitting to the logistic model) is sometimes enhanced with the lag phase understood as time with no growth (Reding-Roman et al. 2017), the Baranyi (fitting to the Baranyi model) assumes suboptimal growth in the lag phase (Baranyi et al. 1993, Baranyi and Roberts 1994).

Note that within this publication ‘method’ refers to the way in which the lag phase is determined, while ‘model’ refers to a set of equations that describe the entire microbial growth curve. For example, a theoretical growth curve can be simulated with a logistic model (where lag is one of the predefined parameters) and later this growth curve can be analysed with different lag calculation methods to estimate its lag phase length.

### How the method's assumptions impact the lag phase estimation results?

Having knowledge and understanding of principles and methodologies adopted to lag phase estimation, it is understood that embedded assumptions in the lag estimation methods will impact the results (Smug et al. 2024). It would be interesting to test different methods to compare their accuracy and robustness in representing microbial growth dynamics. We decided to explore these concepts using empirical and simulated datasets and different lag duration calculation methods. Technical details of the empirical data generation are provided in an accompanying dataset (Opalek 2025) and supplementary materials attached.

## Testing methods for lag phase duration determination on the model-simulated dataset

Given that the methods of calculating the lag phase duration are based on theoretical concepts, we first simulated microbial growth curves based on various well-known deterministic mathematical models: (i) the time-delayed exponential model, which assumes that microbes neither grow nor divide for some time (lag) and then start growing exponentially with a constant growth rate; (ii) the time-delayed logistic model, which assumes that the microbes neither grow nor divide for some time (lag) and then start growing exponentially with a decreasing growth rate; (iii) the time-delayed Monod model where microbes neither grow nor divide for some time (lag) and then the growth rate is coupled with the concentration of resources, and (iv) Baranyi model, which assumes that cells grow and divide in the lag phase but that growth is slower (suboptimal) than in the exponential phase (the adjustment function within the model formula slows down the initial growth but it does not pause it during the lag time). This validation shows how different theoretical concepts fit to one another, e.g. whether the tangent method can robustly determine the duration of the lag phase for the dataset simulated with the logistic model. For each simulated growth curve, the duration of the lag phase was specified to be 2.5 h. See the supplementary materials for the formulation of each of the models.

**Figure 1.**
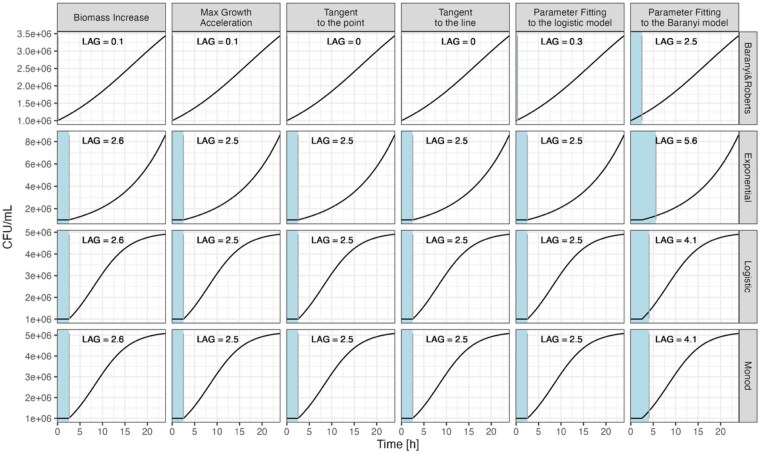
Lag phase durations (blue-coloured areas, vertical lines indicate the end of the lag phase) calculated for data (black lines) simulated under various models (rows) and calculated by different methods (columns). For each simulated growth curve, the duration of the lag phase was specified to be 2.5 h.

Subsequently, we estimated the lag phases using all methods described in the section ‘How to detect the end of the lag phase? Assumptions and models’ (i.e. biomass increase, max growth acceleration, tangent to the point, tangent to the line, parameter fitting to the logistic model, and parameter fitting to the Baranyi model). The calculated lag phases generally agreed well with the true value of 2.5 h and demonstrated consistency across the various methods with one notable exception for the Baranyi model (Fig. 1). Lag phases from curves simulated using the Baranyi model (Fig. 1, first row, Baranyi and Roberts) were not accurately calculated by methods that were not based on the Baranyi model. Conversely, when using the Baranyi method for lag phase estimation (Fig. 1, last column, parameter fitting to the Baranyi model), the calculated lag times were overestimated for curves simulated from models other than the Baranyi model. This discrepancy arises from the fact that these models are based on different conceptualizations of the lag phase. Specifically, the biomass increase and parameter fitting to the logistic model methods assume the lag phase to be the time during which cells do not divide at all, while parameter fitting to the Baranyi model method assumes that cells divide during the lag phase but at a suboptimal growth rate. The Baranyi model additionally assumes a specific adjustment formula for how the growth rate approaches its maximum. In scenarios where cells do not divide initially and then begin dividing at the maximum growth rate—as described by exponential, logistic, and Monod models—the biomass increase method, parameter fitting to the logistic model, tangent method, and max growth acceleration method accurately determine the lag duration. However, the Baranyi model overestimates lag phase duration in these cases because it does not accommodate an abrupt increase in growth rate from zero to maximum, which violates the model's assumptions.

## Empirical dataset: standard, atypically shaped, and no-lag growth curves

Given the discrepancy in the understanding of lag phase, namely (i) the time when cells don't divide at all or (ii) the time when cells may divide but with some suboptimal growth rate, we sought to verify which approach best resembles empirical data under several biological scenarios. To check this, we used three empirical datasets: (A) fresh culture grown in optimal conditions to obtain a standard growth curve, (B) atypically shaped growth curves obtained from cultures starved under various conditions, and (C) curve with no lag (i.e. the shortest lag we can observe) (Opalek 2025). Testing on a variety of curve shapes is crucial to ensure that conclusions drawn are not biased by specific growth conditions. It also allows for a more robust validation of theoretical models across realistic biological variability.

### (A) Standard growth curve

**Figure 2.**
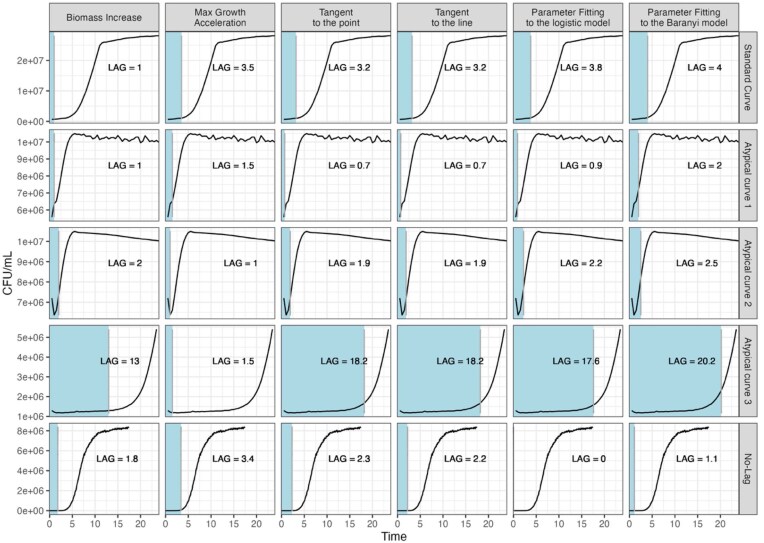
Comparison of lag phase duration (blue-coloured areas, vertical lines indicate the end of the lag phase) estimated by various methods (columns) on empirical dataset (rows). There are three biological contexts tested: (A) standard growth curve (fresh culture grown in optimal conditions, first row), (B) atypically shaped growth curves obtained from starved cultures (rows 2–4), and (C) curve with no lag (minimal lag duration, i.e. exponentially growing cultures transferred to new medium, last row).

Results obtained for empirical data show that the lag duration estimates may vary depending on the lag calculation method even for the typically shaped growth curve. In the analysed example, the longest lag phase was estimated by parameter fitting to the Baranyi model (4 h), while the shortest by the biomass increase method (1 h) (Fig. 2, standard curve).

### (B) Atypical curves

Examples of atypical growth curves include: a very short lag phase (atypical curve 1), the biomass drop at the beginning of the measurements (atypical curve 2), and a very long and turbulent lag phase (atypical curve 3) (Fig. 2, atypical curves 1–3). The biomass drop has little effect on the agreement between the lag estimation methods, in particular the mean lag phase duration was estimated to be 1.9 ± 0.504 h, with the shortest lag estimated by the max growth acceleration method (1 h) and the longest by parameter fitting to the Baranyi model (2.5 h). For the curves with short lags, the lag estimation methods also show rather coherent results, with a mean of 1.13 ± 0.52 h, and the shortest lag estimated by both tangent methods (0.7 h) and the longest by parameter fitting to the Baranyi model (2 h). The largest deviation between methods was observed for the growth curve with a very long lag phase. The parameter fitting to the Baranyi model estimated the longest lag phase of 20.2 h, while the max growth acceleration method mistook noise in the data for the end of the lag phase and estimated a lag phase of only 1.5 h. The mean lag phase duration estimate for this curve is 14.7 ± 6.93 h, but removing the max growth acceleration method, which picked the wrong point, significantly reduces the discrepancy (17.4 ± 2.67 h).

### (C) Curve with no lag

To obtain the growth curve with minimal lag phase duration, i.e. where population biomass constantly increases since the beginning of the measurements, exponentially growing cells were transferred to new growth medium (see supplementary material for details). Indeed, it can be visually assessed that the obtained growth curve does not have a lag in the sense of a nongrowing phase (Fig. 2 no lag, Supplementary Fig. 1: bottom panel); nevertheless, all methods except parameter fitting to the logistic model identified the presence of the nonzero lag phase. Interestingly, the Baranyi model describes better the beginning of the curve than the logistic model, and indeed, this growth curve has a lag phase as understood by Baranyi (growth at a suboptimal rate) (Supplementary  Fig. 1: bottom panel). However, the later phases of this growth curve are better described by the logistic model (Supplementary  Fig. 1: top panel).

The actual experimental curves may not fit perfectly to any of the models which may make it difficult to choose one lag calculation method. Indeed, we show that the real growth curves may yield quite different results for each method and the lags may be composed of two components: (i) time when cells do not divide at all and (ii) later time when they divide with the suboptimal growth rate. An additional difficulty is that in the experimental data there may be some noise in measurements (stochastic errors or variations in measurements that interfere with the underlying patterns), and the effect of such noise has not been studied before. Noisy data points can be either excluded for lag length estimation if they are within the SP or the entire curve may be smoothened, e.g. by using the Tukey smoothening algorithm. These preprocessing techniques may help to reduce the discrepancy between lags calculated by multiple methods (Supplementary Fig. 2).

## Testing the sensitivity of lag determination methods to data noisiness

To understand the source of possible errors and biases in lag calculation on experimental data, we simulated growth curves similar to the empirical one shown in the bottom row of Fig. 2 (no lag).

The curves were simulated from the logistic model; therefore, within this validation, the lag is understood as a time when cells do not divide. Next, to each curve we added noise simulated from the normal distribution with mean = 0 and standard deviation dependent on the initial biomass B0 (Supplementary Fig. 3). Thus, our simulated growth curves could be described as follows:

${\textit{B}_{\rm {noisy}}}( t ) = {\textit {B}}( t ) + N( {0,\ sd*\textit {B}( 0 )} )$ , where *B*(*t*) is the solution from the deterministic logistic model with a lag component (see supplement for the formulation of this model).

**Figure 3.**
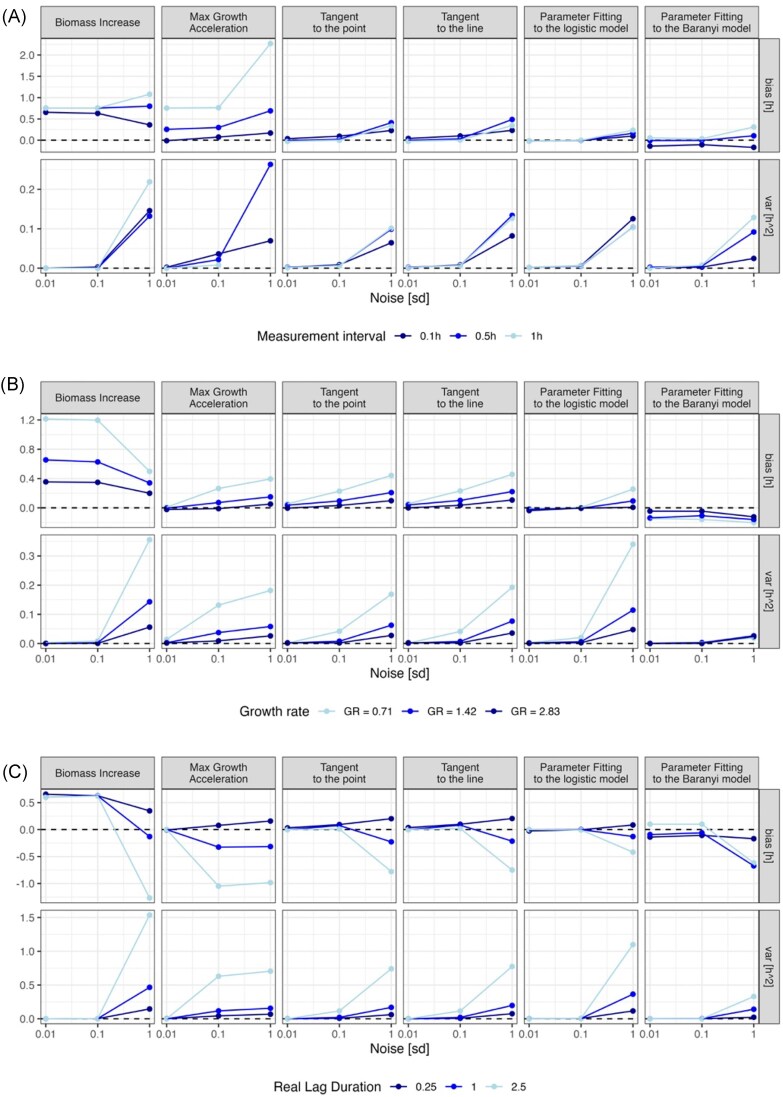
The impact of (A) time intervals between subsequent measurements of population size, (B) growth rate, and (C) real lag duration on lag phase duration estimations. For each sd (noisiness, *x*-axis), lag calculation method (columns), and measurement frequency/growth rate/real lag duration (colours), 500 simulations were conducted (500 growth curves). The *y*-axis illustrates two measures of estimation quality: bias, i.e. the mean difference between estimated and real lag (top row), and precision, i.e. the variance of estimated lag values (bottom row).

We varied the level of noisiness together with other parameters such as time interval between data points (i.e. frequency of measurements, Fig. 3A: 0.1, 0.5, and 1 h), population growth rate (Fig. 3B: 0.71, 1.42, and 2.83), and real lag time (Fig. 3C: 0.25, 1, and 2.5 h). For each combination, we simulated 500 curves. Then, for each of these curves, we estimated the lag length with each of the lag calculation methods and we determined the method bias, i.e. the mean difference between observed and expected lag, and precision, i.e. the variance of estimated values.

First, we tested how the 'frequency of population biomass measurement' impacts the lag length estimation (Fig. 3A). In agreement with previous reports (Baty and Delignette-Muller 2004), it turned out that frequent measurements improve the quality of lag duration estimation. It is especially pronounced in the biomass increase and max growth acceleration methods, where long time intervals between measurements result in higher bias and variance of the estimated values (i.e. the lags tend to be overestimated and the estimates are not precise). Such bias results from the fact that these methods operate only on the data points provided, and they do not use any implicit models to interpolate between them. Specifically, these two methods could detect the correct lag (0.25 h) either at the time point 0 h from the start of measurements (underestimation) or 1 h after the beginning of measurements (overestimation). Additionally, if data are noisy, these methods may either detect the lag phase end at a random noisy point (biomass increase) or classify the entire growth curve as lag since the growth does not accelerate (max growth acceleration). The tangent method is affected to a lesser extent (starts overestimating the lag when there is a high amount of noise), and the parameter fitting to the logistic model is only marginally affected.

Next, we checked whether the population's ‘growth rate’ has an impact on lag estimation (Fig. 3B). We calculated lags for three growth rates representing slow, moderate, and fast growth, and for varied levels of data noisiness. For most of the methods, slow growth results in higher bias and higher variance (i.e. low precision) of lag estimation especially when the data are noisy (Fig. 3B). These results are consistent with our experience, namely, when the growth curve is flat, it is challenging to find a point where the population starts growing exponentially. The biomass increase method tends to overestimate lag duration even in the absence of noise and for cultures growing with a high growth rate (this is due to an insensitive threshold). Max growth acceleration and both tangent methods are sensitive to growth rates to a similar degree. Parameter fitting to the models performs the best for slowly growing cultures especially if the data noisiness is low or moderate.

Another factor that affects the lag length estimation quality is the actual ‘length of the lag’ phase. As the data are simulated with the logistic model, in this case, the lag is assumed to be time when there is no growth (Fig. 3C). While the original data were simulated for a very low lag length to mimic the empirical data from Fig. 2, we varied this parameter in the model. We then observed that the longer the lag, the easier it is to erroneously detect some noise during the lag phase as the first signs of growth. This effect is most pronounced in biomass increase and max growth acceleration methods which shows the biggest biases for long lag lengths (2.5 h). The least affected method is parameter fitting to the logistic model, which shows the most unbiased estimations; however, its precision drops with increasing noise (Fig. 3C).

## Conclusions

Lag phase duration is an important fitness component for microbial populations. Within this publication, we discussed the most popular approaches to calculate the lag phase duration for population-level data. All these methods are developed based on typically shaped growth curves (Baty and Delignette-Muller 2004). Therefore, we implemented and tested them on simulated and empirical data, including atypical curves to show that results may vary. We have highlighted how different understandings of the lag phase, namely (i) time when cells do not divide at all or (ii) time until population reaches optimal growth rate, can drive the choice of the lag estimation method and impact final outcomes. We examined how these different methods adapt to various biological scenarios and how the distinct definitions of lag phase influence the analysis. To further compare the methods in terms of their precision and biases, we simulated growth curves with a varied level of noise and other parameters such as growth rates, lag lengths, or the time intervals between data points (understood as the frequency of measurements). For each of these growth curves, we estimated the lag duration with each of the lag calculation methods.

A key decision while considering the choice of method lies in the biological context—whether, for a specific study, it is more appropriate to define the lag phase as the time when the population does not grow or as the time required to reach the optimal growth rate. Methodological and instrumental limitations must also be considered. For instance, spectrophotometry is the most used method to obtain growth curves. However, this technique has its challenges, such as the detection limit, which can be particularly problematic if the culture starts at very low densities. Specifically, if the inoculum size is small, exponential growth may start before the biomass reaches the OD detection level, and thus, the observed lag duration may be overestimated. Additionally, spectrophotometry measures biomass rather than the number of cells, thus may be also affected by dead or lysed cells or even by the cell shape which may change during the lag phase (Baty et al. 2002).

Although our study focused on a single strain of the model species *Saccharomyces cerevisiae*, we believe that this does not substantially limit the applicability of our conclusions. Rather than varying microbial species or strains, we introduced biologically distinct scenarios that resulted in a diverse range of growth curve shapes, capturing key variations typically observed across different microorganisms. The experimental data are also complemented by *in silico* simulations, i.e. 500 curves per scenario, resulting in a total of 4500 unique growth profiles. This approach allowed us to test the robustness of our analysis framework under varying environmental conditions.

Within our validation study, most of the methods showed similar results, especially for data with typically shaped growth curves. This is in line with the previous observation that the choice of a method influences the calculated lag phase duration to a lesser extent than the data quality and characteristic (Baty and Delignette-Muller 2004). To further improve consistency between methods various preprocessing techniques can be applied, such as smoothening the curve to reduce noise (Supplementary Fig. 2).

A parameter that can significantly improve the quality of analysis and is easily controllable by the experimenter is the frequency of measurements (Fig. 3A). We recommend taking measurements at intervals of no more than 0.5 h and more frequently if an atypical growth curve shape is expected, such as in populations that have experienced environmental stress, toxins, or prolonged starvation. In our analyses on simulated dataset, when measurements were spaced every 1 h, we observed a greater variance between the results than when measurements were spaced every 0.1 h. This high variance was particularly evident for the biomass increase and max growth acceleration methods—both operate only on the datapoints provided and cannot interpolate between measurements to estimate lag duration. This highlights the importance of frequent sampling to ensure accurate and reliable lag phase estimation, particularly for methods sensitive to the intervals between data points.

Another important takeaway is that for growth curves for which lag length estimation is challenging (i.e. long lags, slow growth, and high level of noise) the methods based on model fitting show the lowest bias. When fitting a model to the data, the curve should be truncated after several measurements in the SP because very long and flat curves do not fit models well and can disrupt the entire fitting procedure.

The results presented here emphasize that while determining lag phase duration, all steps (preprocessing, choice of a method, and parameters) influence the lag estimation. We would like to highlight that all these steps should be thoughtfully reported to ensure data reproducibility and credibility. Even the simplest tangent method requires specifying some details such as (i) whether the initial biomass is represented by the first measurement or the minimal value or (ii) the number of data points (or time frame) taken to draw the tangent line—whether a single point (e.g. Moreno-Gámez et al. 2020) or multiple points from the exponential phase (e.g. Jasnos et al. 2008) were used.

This work provides a clearer understanding of how theoretical assumptions and models translate into practical estimations of lag phase duration. Our outcomes highlight the need to align lag phase estimation methods with the biological context and data quality. No single method is universally better than others, rather, understanding the assumptions and limitations of each method is critical to drawing biologically meaningful conclusions about microbial growth dynamics and fitness.

## Supplementary Material

foaf033_Supplemental_File

## References

[bib1] Abi Assaf J, Holden ER, Trampari E et al. Common food preservatives impose distinct selective pressures on *Salmonella typhimurium* planktonic and biofilm populations. Food Microbiol. 2024;121:104517. 10.1016/j.fm.2024.104517. 38637079

[bib2] Adkar BV, Manhart M, Bhattacharyya S et al. Optimization of lag phase shapes the evolution of a bacterial enzyme. Nat Ecol Evol. 2017;1:1–6. 10.1038/s41559-017-0149. 28812634 PMC5640271

[bib3] Baranyi J, McClure PJ, Sutherland JP et al. Modeling bacterial growth responses. J Ind Microbiol. 1993;12:190–4. 10.1007/BF01584189.

[bib4] Baranyi J, Roberts TA. A dynamic approach to predicting bacterial growth in food. Int J Food Microbiol. 1994;23:277–94. 10.1016/0168-1605(94)90157-0. 7873331

[bib5] Baty F, Delignette-Muller ML. Estimating the bacterial lag time: which model, which precision?. Int J Food Microbiol. 2004;91:261–77. 10.1016/j.ijfoodmicro.2003.07.002. 14984774

[bib6] Baty F, Flandrois JP, Delignette-Muller ML. Modeling the lag time of *Listeria monocytogenes* from viable count enumeration and optical density data. Appl Environ Microb. 2002;68:5816–25. 10.1128/AEM.68.12.5816-5825.2002. PMC134405 12450800

[bib7] Bertrand RL. Lag phase is a dynamic, organized, adaptive, and evolvable period that prepares bacteria for cell division. J Bacteriol. 2019;201:1–21. 10.1128/JB.00697-18. PMC6416914 30642990

[bib8] Bheda P. Metabolic transcriptional memory. Molecular Metabolism. 2020;38:100955. 10.1016/j.molmet.2020.01.019. 32240621 PMC7300383

[bib9] Brejning J, Arneborg N, Jespersen L. Identification of genes and proteins induced during the lag and early exponential phase of lager brewing yeasts. J Appl Microbiol. 2005;98:261–71. 10.1111/j.1365-2672.2004.02472.x. 15659180

[bib10] Brejning J, Jespersen L, Arneborg N. Genome-wide transcriptional changes during the lag phase of *Saccharomyces cerevisiae*. Arch Microbiol. 2003;179:278–94. 10.1007/s00203-003-0527-6. 12632260

[bib11] Brejning J, Jespersen L. Protein expression during lag phase and growth initiation in *Saccharomyces cerevisiae*. Int J Food Microbiol. 2002;75:27–38. 10.1016/S0168-1605(01)00726-7. 11999115

[bib12] Buchanan RL, Cygnarowicz ML. A mathematical approach toward defining and calculating the duration of the lag phase. Food Microbiol. 1990;7:237–40. 10.1016/0740-0020(90)90029-H.

[bib13] Cerulus B, Jariani A, Perez-Samper G et al. Transition between fermentation and respiration determines history-dependent behavior in fluctuating carbon sources. eLife. 2018;7:e39234. 10.7554/eLife.39234. 30299256 PMC6211830

[bib14] Chaturvedi K, Basu S, Singha S et al. Predictive microbial growth modelling for an effective shelf-life extension strategy of Chhana (Indian cottage cheese). Food Control. 2023;149:109697. 10.1016/j.foodcont.2023.109697.

[bib15] Cucinotta CE, Dell RH, Braceros KCA et al. RSC primes the quiescent genome for hypertranscription upon cell-cycle re-entry. eLife. 2021;10:1–24. 10.7554/eLife.67033. PMC8186906 34042048

[bib16] Dalgaard P. Modelling of microbial activity and prediction of shelf life for packed fresh fish. Int J Food Microbiol. 1995;26:305–17. 10.1016/0168-1605(94)00136-T. 7488526

[bib17] Danquah MK, Forde GM. Development of a pilot-scale bacterial fermentation for plasmid-based biopharmaceutical production using a stoichiometric medium. Biotechnol Bioproc E. 2008;13:158–67. 10.1007/s12257-007-0080-2.

[bib18] Fridman O, Goldberg A, Ronin I et al. Optimization of lag time underlies antibiotic tolerance in evolved bacterial populations. Nature. 2014;513:418–21. 10.1038/nature13469. 25043002

[bib19] Fu Z, Leighton J, Cheng A et al. Optimization of a *Saccharomyces cerevisiae* fermentation process for production of a therapeutic recombinant protein using a multivariate bayesian approach. Biotechnol Progr. 2012;28:1095–105. 10.1002/btpr.1557. 22581591

[bib20] Hamill PG, Stevenson A, McMullan PE et al. Microbial lag phase can be indicative of, or independent from, cellular stress. Sci Rep. 2020;10:1–20. 10.1038/s41598-020-62552-4. 32246056 PMC7125082

[bib21] Himeoka Y, Mitarai N. When to wake up? The optimal waking-up strategies for starvation-induced persistence. PLoS Comput Biol. 2021;17:1–22. 10.1371/journal.pcbi.1008655. PMC7904209 33571191

[bib22] Jasnos L, Tomala K, Paczesniak D et al. Interactions between stressful environment and gene deletions alleviate the expected average loss of fitness in yeast. Genetics. 2008;178:2105–11. 10.1534/genetics.107.084533. 18430936 PMC2323800

[bib23] Jomdecha C, Prateepasen A. Effects of pulse ultrasonic irradiation on the lag phase of *Saccharomyces cerevisiae* growth. Lett Appl Microbiol. 2011;52:62–9. 10.1111/j.1472-765X.2010.02966.x. 21143488

[bib24] Lee H-YY, Cheng K-YY, Chao J-CC et al. Differentiated cytoplasmic granule formation in quiescent and non-quiescent cells upon chronological aging. Microb Cell. 2016;3:109–19. 10.15698/mic2016.03.484. 28357341 PMC5349021

[bib25] Liu Z, Fels M, Dragone G et al. Effects of inhibitory compounds derived from lignocellulosic biomass on the growth of the wild-type and evolved oleaginous yeast *Rhodosporidium toruloides*. Ind Crops Prod. 2021;170:113799. 10.1016/j.indcrop.2021.113799.

[bib26] Madigan MT, Bender KS, Buckley DH et al. Brock Biology of Microorganisms, 15th edn. Pearson Education, 2018.

[bib27] Martinez MJ, Roy S, Archuletta AB et al. Genomic analysis of stationary-phase and exit in *Saccharomyces cerevisiae*: gene expression and identification of novel essential genes. MBoC. 2004;15:5295–305. 10.1091/mbc.e03-11-0856. 15456898 PMC532011

[bib28] McEntire KD, Gage M, Gawne R et al. Understanding drivers of variation and predicting variability across levels of biological organization. Integr Comp Biol. 2022;61:2119–31. 10.1093/icb/icab160. 34259842

[bib29] Megur A, Daliri EBM, Balnionytė T et al. In vitro screening and characterization of lactic acid bacteria from Lithuanian fermented food with potential probiotic properties. Front Microbiol. 2023;14:1213370. 10.3389/fmicb.2023.1213370. 37744916 PMC10516296

[bib30] Moreno-Gámez S, Kiviet DJ, Vulin C et al. Wide lag time distributions break a trade-off between reproduction and survival in bacteria. Proc Natl Acad Sci USA. 2020;117:18729–36. 10.1073/pnas.2003331117. 32669426 PMC7414188

[bib31] Opalek M, Smug B, Doebeli M et al. On the ecological significance of phenotypic heterogeneity in microbial populations undergoing starvation. Microbiol Spectr. 2022;10:e00450–21. 10.1128/spectrum.00450-21. PMC8754142 35019773

[bib32] Opalek M. *S.cerevisiae* Growth Curves.txt. figshare. Dataset. 2025, 10.6084/m9.figshare.29137718.

[bib33] Reding-Roman C, Hewlett M, Duxbury S et al. The unconstrained evolution of fast and efficient antibiotic-resistant bacterial genomes. Nat Ecol Evol. 2017;1:0050. 10.1038/s41559-016-0050. 28812723

[bib34] Rolfe MD, Rice CJ, Lucchini S et al. Lag phase is a distinct growth phase that prepares bacteria for exponential growth and involves transient metal accumulation. J Bacteriol. 2012;194:686–701. 10.1128/JB.06112-11. 22139505 PMC3264077

[bib35] Smug BJ, Opalek M, Necki M et al. Microbial lag calculator: a shiny-based application and an R package for calculating the duration of microbial lag phase. Methods Ecol Evol. 2024;15:301–7. 10.1111/2041-210X.14269.

[bib36] Valík Ľ, Šipošová P, Koňuchová M et al. Modelling the effect of temperature on the initial decline during the lag phase of *Geotrichum candidum*. Appl Sci. 2021;11:7344. 10.3390/app11167344.

[bib37] Verbelen PJ, Dekoninck TML, Saerens SMG et al. Impact of pitching rate on yeast fermentation performance and beer flavour. Appl Microbiol Biotechnol. 2009;82:155–67. 10.1007/s00253-008-1779-5. 19018524

[bib38] Vermeersch L, Perez-Samper G, Cerulus B et al. On the duration of the microbial lag phase. Curr Genet. 2019;65:721–7. 10.1007/s00294-019-00938-2. 30666394 PMC6510831

[bib39] Vriesekoop F, Pamment NB. Acetaldehyde addition and pre-adaptation to the stressor together virtually eliminate the ethanol-induced lag phase in *Saccharomyces cerevisiae*. Lett Appl Microbiol. 2005;41:424–7. 10.1111/j.1472-765X.2005.01777.x. 16238646

