# Supplementary material for: Assessing methods for estimating microbial lag phase duration: a comparative analysis using Saccharomyces cerevisiae empirical and simulated data
Source: FEMS Yeast Res. 2025 Jul 11;25:foaf033. doi: 10.1093/femsyr/foaf033 (PMC12258147; doi:10.1093/femsyr/foaf033)
Supplement: foaf033_Supplemental_File [file foaf033_supplemental_file.docx]

Assessing Methods for Estimating Microbial Lag Phase Duration: A Comparative Analysis Using *S.cerevisiae* Empirical and Simulated Data

Opalek M., Wloch-Salamon D., Smug B. J.

Supplementary Materials

**Supplementary Information**

The code used to simulate growth curves and fit the lag phase duration can be found here:

<https://github.com/bognabognabogna/lag_calc_methods_comparison> (Release v1.0.0)

**Empirical growth curves acquisition**

Growth curves were acquired from publicly available dataset [Opalek 2025] and during previous research projects [Opalek, Smug, Doebeli, and Wloch-Salamon 2022].

**Simulated growth curves**

Growth curves shown in Fig. 1 were simulated from the models described in the Supplementary Materials, and for time points spanning every 6 minutes between 0 and 24 hours. The parameters used are listed in the Sup. Tab. 2.

Growth curves with noise analysed in Fig. 3 were simulated using parameters described in the Supplementary Material.

**Lag duration calculation methods**

Lag duration was calculated using R package *miLAG* version 1.0.5 [Smug, Opalek, Necki, and Wloch‐Salamon 2024] with default parameters: biomass increase threshold = 10^3, initial inoculum size equal to first observation, 3 points included in fitting line to max growth rate in tangent method. For model parameters check the Supplementary Material.

**Data processing and visualization**

Data processing and graphs generation was conducted with R version 4.4.2, using the following packages: *miLAG* [Smug, Opalek, Necki, and Wloch‐Salamon 2024], *ggplot2* [Wickham 2016]*, dplyr* [Wickham, François, Henry, and Müller 2022]*, readxl* [Wickham and Bryan 2022]*, tidyr* [Wickham, Vaughan, and Girlich 2023]*, DEoptim* [Mullen, Ardia, Gil, Windover, and Cline 2011]*, deSolve* [Soetaert, Petzoldt, and Setzer 2010]*, scales* [Wickham, Pedersen, and Seidel 2023]*, data.table* [ Barrett 2025]*, nlsMicrobio* [Baty and Delignette-Muller 2013]*, patchwork* [Pedersen 2025]*.*

**Supplementary Figures**

Frequent biomass measurements facilitate precise estimation of lag duration


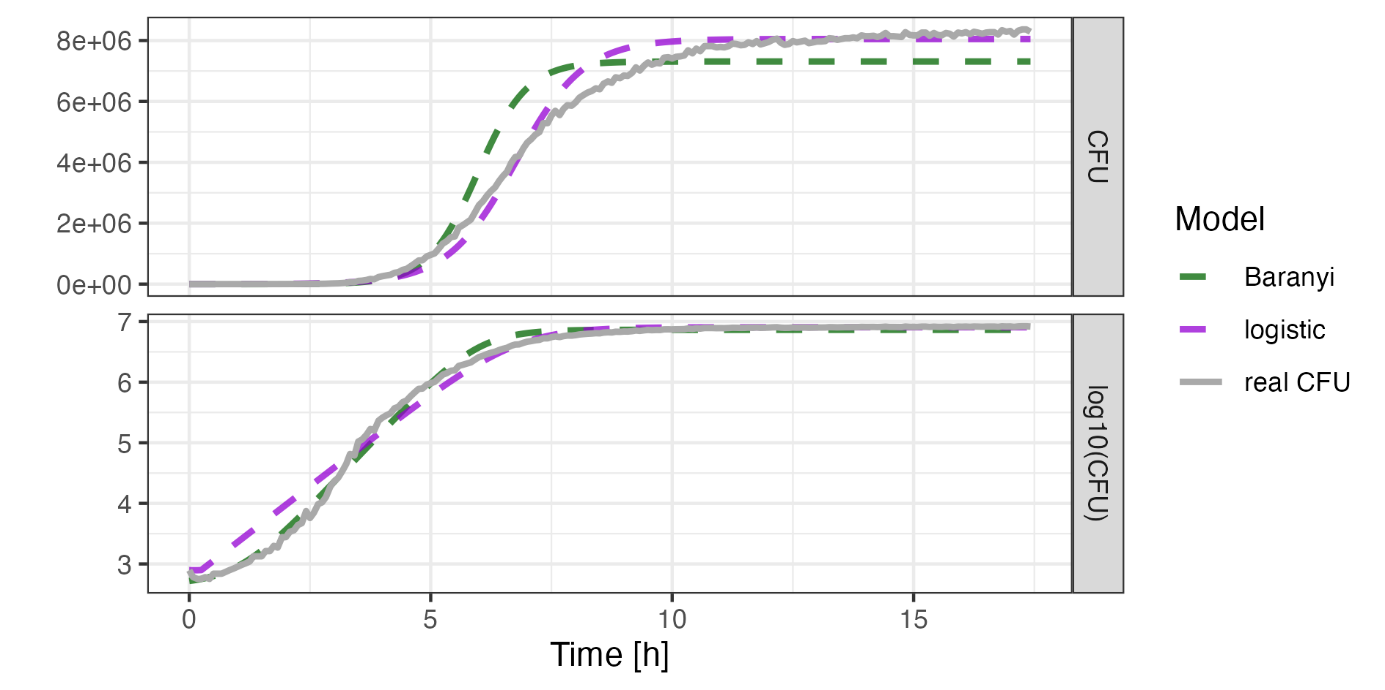


Sup. Fig. 1. *No-lag Empirical Curve* (grey line) fitted to logistic (violet line) and Baranyi (green line) models shown on normal (top panel) and logarithmic (bottom panel) scales. The Baranyi model describes the beginning of the curve better than the logistic model (which can be seen on the log scale), although it performs worse later in the growth phase (as seen on the normal scale).


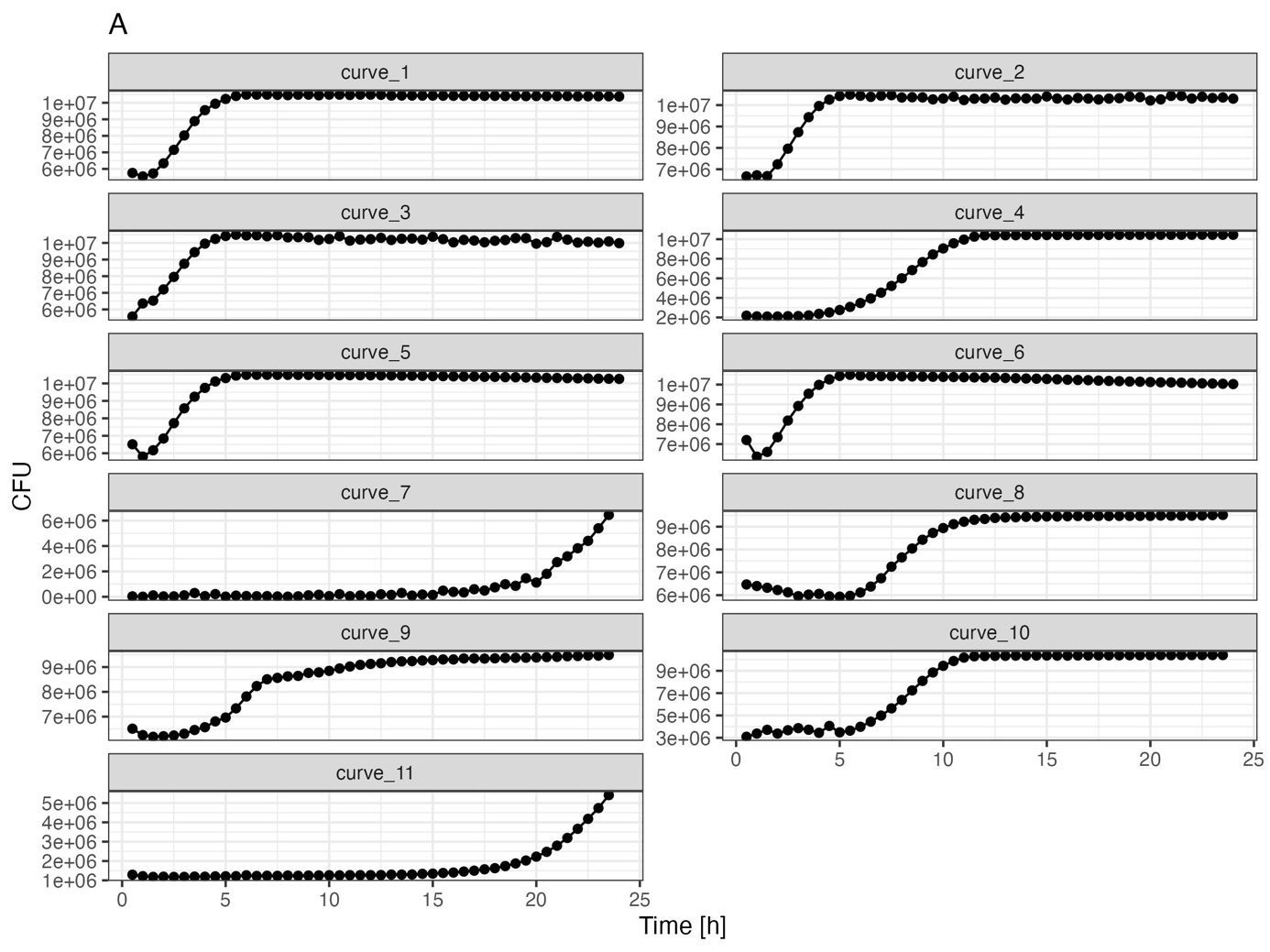


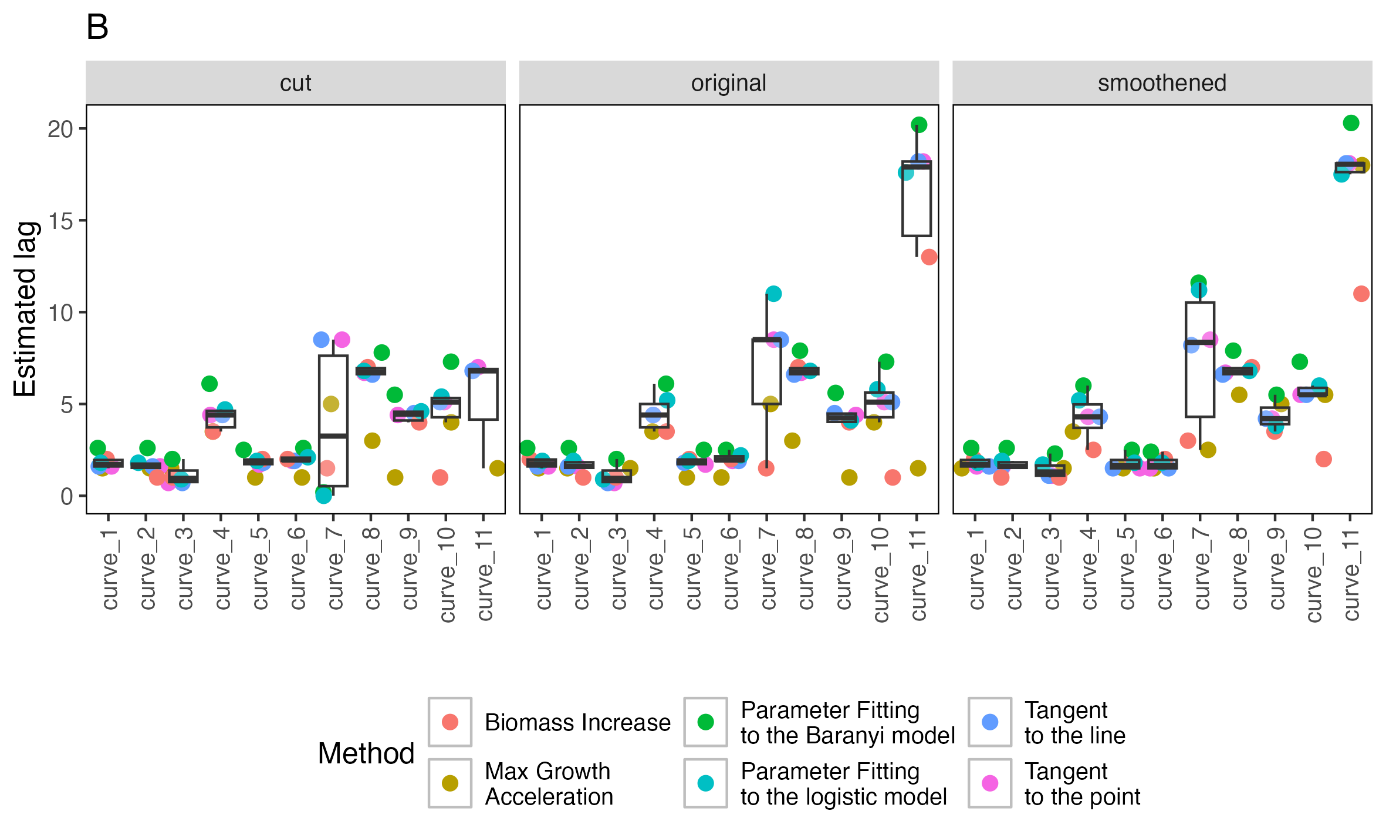


Sup. Fig. 2. (A) empirical curves. (B) Distribution of lag duration estimated by six discussed methods (color) per each curve shown in panel A (panel B: x-axis) and pre-processing algorithm (column). “Original” means no pre-processing has been applied, “cut” means the data curve has been shortened to 12 hours only, and “smoothened” means Tukey smoothening has been applied.


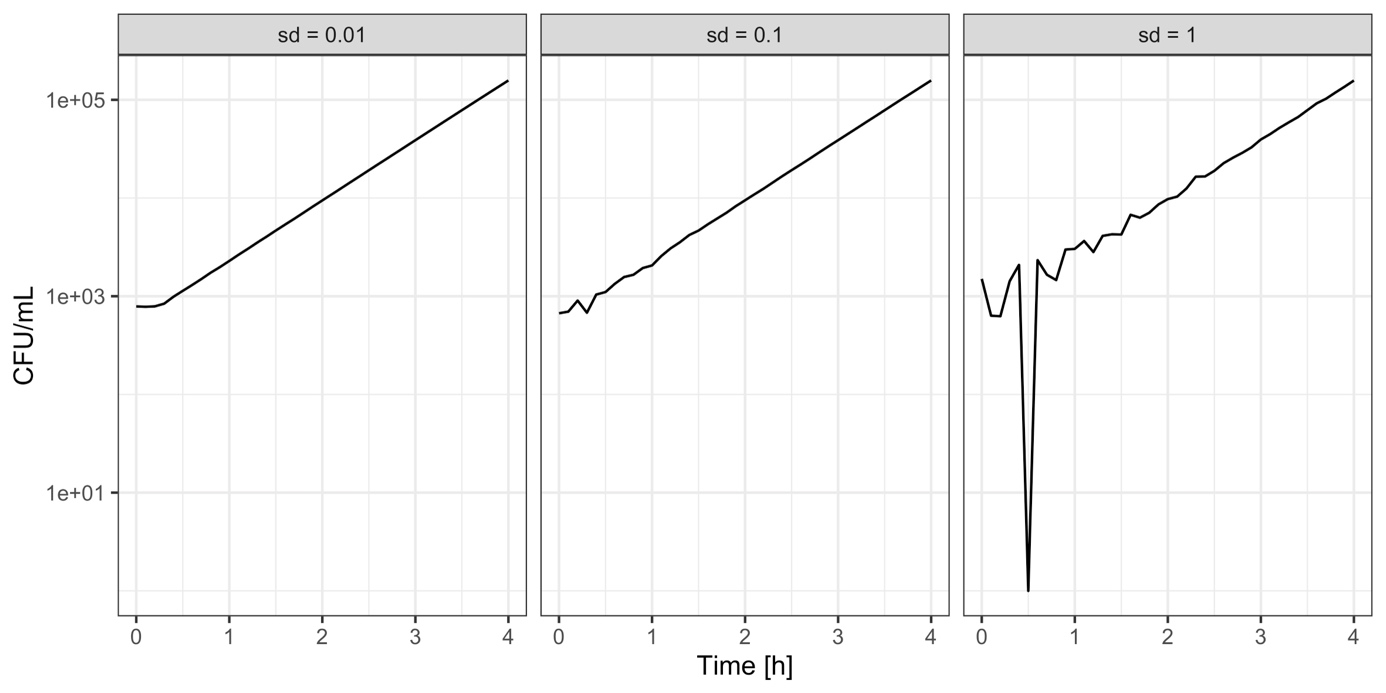


Sup. Fig. 3. The visualization of the impact of introduced noisiness (sd) on growth curve shape (initial 4 hours). The noise was simulated from the random distribution with mean = 0 and standard deviation standardized by the initial biomass N0.

**Atypically shaped growth curves acquired form starved populations.**

Growth curves (Fig. 2 *Atypical curves* & Sup. Fig. 2) were acquired by growing haploid, prototroph *Saccharomyces cerevisiae* S288C strain in rich YPD (yeast peptone-dextrose) medium at 30^o^C. The populations were starved before the growth procedure. The populations’ densities were monitored by absorbance measurements (optical density (OD), 600 nm in SpectraMax iD3) taken every half an hour. The biomass was calculated according to the equations:

For OD measurements below 0.1: 10^(1.885845 + 28.72096×OD)^

For OD measurement equal or above 0.1: 3566518×OD + 26754296×OD^2^ -19881567×OD^3^

Sup. Tab. 1. The summary of starvation conditions, chronological age, and phenotypic states of populations used to acquire growth curves analysed. The data was originally collected to analyse possible advantages of phenotypic heterogeneity within a starving clonal population and it was used in Opalek *et al.* 2022. The data was not analysed nor visualised in the form presented in this publication.

| CURVE ID | STARVATION MEDIA | STARVATION DURATION | CELL TYPE |
| --- | --- | --- | --- |
| Atypical Curve 1 | Spent YPD medium | 7 days | natural Q+NQ mix culture |
| Atypical Curve 2 | Spent YPD medium | 14 days | Q monoculture |
| Atypical Curve 3 | H_2_O | 42 days | NQ monoculture |
| curve_1 | Spent YPD medium | 7 days | NQ monoculture |
| curve_2 | Spent YPD medium | 7 days | NQ monoculture |
| curve_3 | Spent YPD medium | 7 days | natural Q+NQ mix culture |
| curve_4 | H2O | 14 days | NQ monoculture |
| curve_5 | Spent YPD medium | 14 days | Q monoculture |
| curve_6 | Spent YPD medium | 14 days | Q monoculture |
| curve_7 | H2O | 35 days | NQ monoculture |
| curve_8 | Spent YPD medium | 35 days | NQ monoculture |
| curve_9 | Spent YPD medium | 42 days | NQ monoculture |
| curve_10 | H2O | 42 days | Q monoculture |
| curve_11 | H2O | 42 days | NQ monoculture |

**Formulations of mathematical models used to simulate the growth curve data:**

1. Exponential model

$$\frac{dN}{dt}=\left\{ \begin{aligned} rN, t\geq L \\ 0, t<L \end{aligned} \right.$$

which can be solved as

$$N(t)=\left\{ \begin{aligned} N\left( 0 \right)e^{r(t-L)}, t\geq L \\ N(0), t<L \end{aligned} \right.$$

where N(t) is the population size at time t, L is the lag phase duration, and r is the growth rate (constant in time).

1. Logistic model

$$\frac{dN}{dt}=\left\{ \begin{aligned} rN\left( 1-\frac{N}{K} \right), t\geq L \\ 0, t<L \end{aligned} \right.$$

where N(t) is the population size at time *t*, *L* is the lag phase duration*, r* is the growth rate (constant in time), and *K* is the saturation constant (i.e. the maximum population size).

1. Monod Model

$$\frac{dN}{dt}=\left\{ \begin{aligned} a\frac{VG}{K^{G}+G}N, t\geq L \\ 0, t<L \end{aligned} \right.$$

$$\frac{dG}{dt}=\left\{ \begin{aligned} -\frac{VG}{K^{G}+G}G, t\geq L \\ 0, t<L \end{aligned} \right.$$

where N(t) is the population size at time t, G(t) is the limiting nutrient (for example glucose) concentration at time *t*, *L* is the lag phase duration. V denotes the maximal rate of the glucose uptake pathway, and $K^{G}$ denotes the Michaelis-Menten constant (so that if $K^{G}$=G, $\frac{VG}{K^{G}+G}$ = V/2). Moreover, the efficiency of converting glucose into biomass is described by a parameter a, which for simplicity is assumed to be constant.

1. Baranyi and Roberts Model

$$\frac{dN}{dt}=r\left( 1-\frac{N}{K} \right)\frac{Q}{1+Q}N$$

$$\frac{dQ}{dt}=vQ$$

Where N(t) is the population size at time *t*, *K* is the saturation constant (i.e. the maximum population size), and *Q* represents the physiological state proportional to the concentration of some critical substrate.

In practice we used the formula provided by the *nlsMicrobio* package:

$log_{10}N\left( t \right)= log_{10} K+$ ${log}_{10}$ ((-1 + exp($r$L) + exp($r$t))/(exp($r$t) - 1 + exp(rL) * 10^($log_{10} K$ - $log_{10}N\left( 0 \right)$)))

Sup. Table 2. Default parameters used to simulate data from models (shown in Figure 1)

| Parameter name | Default value |
| --- | --- |
| Growth rate $r$ (exponential and Baranyi model) | 0.1 [$h^{-1}$] |
| Max. growth rate $r$(logistic model) | 0.25 [$h^{-1}$] |
| Carrying capacity $K$(logistic model and Baranyi model) | $5*{10}^{6}$ [cells/mL] |
| Initial biomass $N_{0}$ | ${10}^{6}$ [cells/mL] |
| Lag $L$ in logistic, exponential, Baranyi and Monod models | 2.5 [h] |
| Initial resource concentration $G_{0}$ (Monod model) | 13.9 [mmol substrate] |
| Efficiency of substrate uptake in Monod model $a$ | 3*${10}^{5}$ [cells/mmol substrate] |
| Maximal substrate uptake rate in Monod model $V$ | 1.5*${10}^{(-5)}$ [mmol substrate /cells× h]) |
| Michaelis-Menten constant in Monod model $K^{G}$ | 300 [mmol substrate] |

Sup. Table 3. Default parameters used to simulate data in logistic model with noise (shown in Figure 3), These values were obtained by fitting logistic model to the empirical growth curve with no lag, shown in Figure 2. Values shown in this table are rounded to 10^(-2).

| Parameter name | Default value |
| --- | --- |
| Max. growth rate $r$(logistic model) | 1.42 [$h^{-1}$] |
| Carrying capacity $K$(logistic model) | $8049712$[cells/mL] |
| Initial biomass $N_{0}$ | 788.5[cells/mL] |
| Lag $L$ | 0.25 [h] |

**References**

Barrett T, Dowle M, Srinivasan A, Gorecki J, Chirico M, Hocking T, Schwendinger B, Krylov I (2025). data.table: Extension of 'data.frame', https://r-datatable.com.

Baty F, Delignette-Muller M-L. 2013. nlsMicrobio: data sets and nonlinear regression models dedicated to predictive microbiology.

Mullen K, Ardia D, Gil D, Windover D, Cline J. 2011. DEoptim : An R Package for Global Optimization by Differential Evolution. *J. Stat. Softw.*, **40**. http://www.jstatsoft.org/v40/i06/.

Opalek M. 2025. S.cerevisiae Growth Curves.txt. figshare. Dataset. https://doi.org/10.6084/m9.figshare.29137718.

Opalek M, Smug B, Doebeli M, Wloch-Salamon D. 2022. On the Ecological Significance of Phenotypic Heterogeneity in Microbial Populations Undergoing Starvation Cuomo CA. (ed). *Microbiol. Spectr.*, **10**. https://journals.asm.org/doi/10.1128/spectrum.00450-21.

Pedersen T (2025). patchwork: The Composer of Plots.].

Smug BJ, Opalek M, Necki M, Wloch‐Salamon D. 2024. Microbial lag calculator: A shiny‐based application and an R package for calculating the duration of microbial lag phase. *Methods Ecol. Evol.*, **15**: 301–307. https://besjournals.onlinelibrary.wiley.com/doi/10.1111/2041-210X.14269.

Soetaert K, Petzoldt T, Setzer RW. 2010. Solving Differential Equations in R : Package deSolve. *J. Stat. Softw.*, **33**. http://www.jstatsoft.org/v33/i09/.

Wickham H. 2016. ggplot2: Elegant Graphics for Data Analysis. https://ggplot2.tidyverse.org.

Wickham H. 2023. plyr: Tools for Splitting, Applying and Combining Data. http://had.co.nz/plyr.

Wickham H, Bryan J. 2022. readxl: Read Excel Files. https://cran.r-project.org/package=readxl.

Wickham H, François R, Henry L, Müller K. 2022. dplyr: A Grammar of Data Manipulation. https://cran.r-project.org/package=dplyr.

Wickham H, Pedersen LT, Seidel D. 2023. scales: Scale Functions for Visualization. https://scales.r-lib.org.

Wickham H, Vaughan D, Girlich M. 2023. tidyr: Tidy Messy Data. https://cran.r-project.org/package=tidyr.
